# Proteomic Analysis of *Bifidobacterium longum* subsp. *infantis* Reveals the Metabolic Insight on Consumption of Prebiotics and Host Glycans

**DOI:** 10.1371/journal.pone.0057535

**Published:** 2013-02-26

**Authors:** Jae-Han Kim, Hyun Joo An, Daniel Garrido, J. Bruce German, Carlito B. Lebrilla, David A. Mills

**Affiliations:** 1 Department of Food Nutrition, Chungnam National University, Yuseong-gu, Daejeon, Korea; 2 Graduate School of Analytical Science and Technology, Chungnam National University, Yuseong-gu, Daejeon, Korea; 3 Department of Viticulture and Enology, University of California Davis, Davis, California, United States of America; 4 Foods for Health Institute, University of California Davis, Davis, California, United States of America; 5 Department of Food Science and Technology, University of California Davis, Davis, California, United States of America; 6 Department of Chemistry, University of California Davis, Davis, California, United States of America; University of Florida, United States of America

## Abstract

*Bifidobacterium longum* subsp. *infantis* is a common member of the intestinal microbiota in breast-fed infants and capable of metabolizing human milk oligosaccharides (HMO). To investigate the bacterial response to different prebiotics, we analyzed both cell wall associated and whole cell proteins in *B. infantis.* Proteins were identified by LC-MS/MS followed by comparative proteomics to deduce the protein localization within the cell. Enzymes involved in the metabolism of lactose, glucose, galactooligosaccharides, fructooligosaccharides and HMO were constitutively expressed exhibiting less than two-fold change regardless of the sugar used. In contrast, enzymes in N-Acetylglucosamine and sucrose catabolism were induced by HMO and fructans, respectively. Galactose-metabolizing enzymes phosphoglucomutase, UDP-glucose 4-epimerase and UTP glucose-1-P uridylytransferase were expressed constitutively, while galactokinase and galactose-1-phosphate uridylyltransferase, increased their expression three fold when HMO and lactose were used as substrates for cell growth. Cell wall-associated proteomics also revealed ATP-dependent sugar transport systems associated with consumption of different prebiotics. In addition, the expression of 16 glycosyl hydrolases revealed the complete metabolic route for each substrate. Mucin, which possesses O-glycans that are structurally similar to HMO did not induced the expression of transport proteins, hydrolysis or sugar metabolic pathway indicating *B. infantis* do not utilize these glycoconjugates.

## Introduction

*Bifidobacterium longum* subsp. *infantis* (*B. infantis*) is a common member of the gastrointestinal tract (GIT) of breast-fed infants [1]. The establishment of a bifidobacterial-dominant microbiota has received considerable attention regarding the development of the infant [2]. One potential means by which specific bifidobacteria succeed in colonizing the infant GIT is through prebiotic enrichment by human milk oligosaccharides (HMO). Human milk contains a significant amount of oligosaccharides (∼15 g l^−1^), in contrast to bovine or formula milk [3], [4]. HMO is a term that collectively refers to approximately 200 different types of glycans with diverse structures with a length smaller than 20 units of carbohydrates [5], [6]. These HMO are composed of hexoses (Hex) and N-Acetylhexosamines (HexNAc) connected through β1-3 or β1-4-glycosidic linkage with additional decoration of fucose (Fuc) and N-Acetylneuraminic acid (NeuAc). Since the human host does not have the enzymatic capacity to degrade these polymers, HMO are not considered to be metabolized by infant, and have been shown to arrive to the lower GIT [7]. HMO are considered part of the innate immune system being used as decoy binding sites for intestinal pathogens in the developing newborn [8]. Another role is the enrichment of bifidobacteria in the GIT [9]. While *B. infantis* and other bifidobacteria can actively utilize HMO as growth substrate, other intestinal bacteria such as streptococci, enterococci, *E. coli* and *Clostridium* sp. cannot use HMO as a carbon source, emphasizing the bifidogenic potential of HMO [10]. Genome sequencing showed that *B. infantis* has a 40 kb gene cluster, termed HMO cluster I, that encodes several enzymes and transport systems required for HMO catabolism including α-fucosidase, α-sialidase, β-hexosaminidase, β-galactosidase and the ABC transport systems with six family 1, extracellular solute binding proteins (SBPs) predicted to bind oligosaccharides [11]. These clusters of HMO-linked genes suggested a broader evolutionary partnership among *B. infantis*, the developing infant and human milk [12].

While current commercial prebiotics used in infant formulas, such as fructooligosaccharides (FOS) and galactooligosaccharides [13], have been shown to be bifidogenic, these commercial prebiotics do not replicate the many different roles that HMO carry out in the developing infant. Since *B. infantis* displays several mechanistic preferences for HMO consumption, this subspecies represents a useful reference with which to assess the similarity of commercial prebiotics to actual HMO. This in turn will aid in the design of more effective prebiotics for use in infant formulas as well as other conditions where very selective symbiotic applications may be useful.

Global expression analysis of whole cell proteome by LC-MS/MS can provide direct information relative to biological systems such as metabolic pathways and regulatory networks. Despite the advantages of whole cell proteomics, it is still challenging to reliably identify cell wall associated (CWA) proteins due to the fact that these proteins are often sequestered into the insoluble material upon cell lysis. The lack of CWA proteins in proteomic analyses results in significant loss of biological information since membrane proteins or cell wall-anchored proteins also play important roles in transport, exopolysaccharide production, hydrolysis of macromolecules, and sensing of extracellular signals. Several proteomic methods have been developed to specifically identify the CWA proteins, including a 2-D gel based proteomic analysis followed by fractionation [14], [15], [16], or labeling the cell surface proteins via biotinylation followed by affinity capture [17], [18]. An alternative approach is the tryptic digestion of exposed epitopes on cell surface proteins from intact cells in isotonic solution followed by the MS analysis, which often called “shaving and shedding” [19], [20].

In this study, we have investigated the impact of various prebiotics on the proteomic expression profiles of *B. infantis* ATCC 15697. The microorganism was cultured with seven different sugars and prebiotics including HMO and mucin, and more than 500 proteins were quantitatively identified enabling us to explore the entire activity cell in a given substrate. Importantly, we developed a proteomic analysis method that allowed reliable determination of cell wall associated proteins. Through this method, the expression of cell surface/membrane associated proteins can be determined, maximizing the total protein identification per cell. This has allowed a more comprehensive comparison of the *B. infantis* physiology on these prebiotics.

## Materials and Methods

### Cell Culture

*Bifidobacterium longum* subsp. *infantis* ATCC 15697 was obtained from the American Type Culture Collection (Manassas, VA). Cultures were routinely maintained in de Mann-Rogosa-Sharp (MRS) medium with no carbon source, and supplemented with 0.05% w/v L-cysteine (Sigma-Aldrich, St. Louis, MO), and 2% w/v of either lactose (Sigma Aldrich, MO), purified HMO [21], mucin from porcine stomach type III (Sigma Aldrich, MO), FOS (raftilose Synergy 1, Orafti, Malvern, PA), inulin (raftiline HP, Orafti, Malvern, PA) or GOS (Purimune, GTC Nutrition, Golden, CO). These experiments were performed in duplicates. Cells were anaerobically grown in a vinyl chamber (Coy Laboratory Products, Grass Lake, MI) at 37°C for 24 h, in an atmosphere consisting of 5% carbon dioxide, 5% hydrogen, and 90% nitrogen. Optical density was assayed using a PowerWave microplate spectrophotometer (BioTek Instruments, Inc., Winoosky, VT). Due to the significant high turbidity of medium, cell growth on mucin was evaluated separately. Mucin was autoclaved for 10 minutes and added on the media (50 ml) at a final concentration of 20 g l^−1^. Cell growth was monitored by measuring optical density of cell at 600 nm in a Shimadzu UV1601 spectrophotometer (Shimadzu Scientific Instruments, Columbia MD). Cell cultures supplemented with 2% (w/v) of glucose and 2% (w/v) of HMO were obtained in similar conditions as control experiments.

### Proteomic Sample Preparation

*B. infantis* cells were taken at the exponential phase of growth and normalized at an OD of 1.0 by dilution or concentration. After centrifugation to remove the spent media, 15 mL of cells were washed three times with phosphate buffer saline (PBS) followed by three more washes with urea buffer (8 M Urea/0.1 M Tris, pH 8.5). The cell pellet was resuspended in 600 µL of urea buffer, then mechanically disrupted by silica beads in a bead-beater (FastPrep; QBiogene, Carlsbad, CA, USA) for eight cycles of 30 s pulses and 30 s on ice. Upon centrifugation, the soluble and the insoluble fractions were stored separately at −80°C until further analysis.

For proteome analysis of the soluble fraction, a sample volume containing 200 mg of protein was transferred to a new microcentrifuge tube and precipitated by the addition of ethanol (75% (v/v) final) at −20°C. After centrifugation, the protein pellet was resuspended in 100 µL of 0.1 M Tris/1 M urea buffer (pH 8.5). Proteins were then digested using 5 µg of mass spectrometry grade trypsin (Promega, Madison, WI, USA) overnight at 37°C. In order to obtain the proteome of insoluble fraction, the insoluble cell debris pellet was resuspended in 1 mL of PBS and washed three times with urea buffer (8 M Urea/0.1 M Tris). The resulting pellet was resuspended again in 100 µL of 0.1 M Tris/1M urea buffer (pH 8.5) and digested with trypsin overnight. These two fractions, soluble and CWA, were analyzed independently. The tryptic peptides from two fractions were cleaned independently using a Macro Trap with Peptide concentration and Desalting cartridge (Michrom, Aurburn, CA, USA) according to the manufacturer’s instructions. The resultant peptides were eluted in 98% acetonitrile in water and then dried prior to mass spectrometry analysis.

### Mass Spectrometry Analysis

Peptides were reconstituted in water at concentrations corresponding to between 40 and 200 ng of the original protein per 2 µL injection. Nano LC/MS and nano LC/MS/MS analyses were performed on an Agilent HPLC-Chip Quadrupole Time-of-Flight (Q-TOF) MS system equipped with a microwell plate autosampler (maintained at 6°C), capillary sample loading pump, nano pump, HPLC Chip/MS interface, and Agilent 6520 Q-TOF MS detector. The chip used consisted of a 9×0.075-mm i.d. enrichment column and a 150×0.075-mm i.d. analytical column. For sample loading, the capillary pump delivered 0.1% formic acid in 3.0% acetonitrile/water (v/v) isocratically at 4.0 µL/min. Following sample injection, a nano pump gradient was delivered at 0.4 µL/min using (A) 0.1% formic acid in 3.0% acetonitrile/water (v/v) and (B) 0.1% formic acid in 90.0% acetonitrile/water (v/v). Samples were eluted with 0% B, 0.00–2.50 min; 0 to 16% B, 2.50–10.00 min; 16 to 44% B, 10.00–30.00 min; 44 to 100% B, 30.00–35.00 min; and 100% B, 35.00–45.00 min. This was followed by equilibration at 0% B, 45.01–65.00 min. The drying gas temperature was set at 325°C with a flow rate of 5.0 L/min (2.5 L of filtered nitrogen gas and 2.5 L of filtered dry compressed air).

Single-stage MS spectra were acquired in the positive ionization mode over a mass range of m/z 400–3,000 with an acquisition time of 1 s per spectrum. Mass correction was enabled using reference masses of m/z 322.048, 622.029, 922.010, 1,221.991, and 1,521.971 (ESI-TOF Calibrant Mix G1969-85000, Agilent Technologies, Santa Clara, CA, USA).

### Protein Identification

All MS/MS samples were analyzed by X! Tandem (GPM-XE manager, ver. 2.2.1). X! Tandem was set up to search against the proteome of *B. infantis* ATCC 15697 and common contaminant proteins and searched with a fragment ion mass tolerance of 100 ppm. Oxidation of methionine was specified as a variable modification in X! Tandem. The probability (log(e)) cutoff for peptide assignment was set at −2. For the identification, two conditions were required: (a) more than two unique peptides per protein (*P_uniq_*≥2) and (b) the probability cutoff for protein less than −6 (log(e)≤−6). Additional algorithms for protein identification validated the protein identification results by X! Tandem. All proteins identified by the X! Tandem were also assigned by the peptide prophet [22] and protein prophet algorithm [23] with the probability greater than 95% and 99% respectively. Since the experiments were performed on biological duplicates an additional rule was applied for the decision of protein expression. If a protein was identified in one of the duplicates with high probability (*P_uniq_*≥2 and log(e)≤−6) then it was considered expressed. If a protein had a log(e) value between −6 and−4 and a *P_uniq_*≥2 but was found in both duplicates, it was also considered as expressed.

### Protein Quantification

Relative amounts of protein in a sample were calculated by the spectral counting method [24] using the proteome on lactose grown cells as control. Normalized spectral abundance factor (NSAF), which represented the relative abundance of protein in a sample, was estimated from the total number of spectra used for the identification of protein (*SpC*) and the number of aminoacids of the protein (*L*). For accurate estimations, the relative amount of protein was calculated only if the protein NSAF value was higher than 1.0 or *SpC* of both proteins were ≥6.

### Determination of Protein Localization

For this purpose, a scoring system was introduced that employed the NSAF values of each protein in the soluble and insoluble fraction (Table S1). If the protein was observed only at the insoluble fraction, or the NSAF value of that protein in the insoluble fraction was more than 3-fold higher than the NSAF value of the same protein in the soluble fraction, the protein had score +1. If the NSAF ratio was between 2- and 3-fold, a protein was given a score of +0.5. When the NSAFs values of the same protein in the soluble and insoluble fractions were within 2-fold difference, the protein was given a score of zero. If the NSAF of a protein at the soluble fraction was larger than that of at insoluble fraction, the scores were attributed using the same criteria above, however the values were negative. Once scores were determined, values from the experiments with six different carbon sources were averaged. If a protein had an average score between +0.5 to +1.0 it was classified as a CWA protein. If the average score of a protein was between −0.5 to −1.0, it was considered as cytosolic (CYT) protein (Table S2).

## Results

### Cell Growth and Overall Protein Expression Profiles

*B. infantis* was able to utilize various mono and oligosaccharides as growth substrates. The specific cell growth rates on GOS, FOS, inulin, and HMO were in a range of 0.14∼0.19 (hr^−1^) which are similar to that of glucose and lactose (Figure S1A). Mucin is a large extracellular protein that is heavily glycosylated. The O-glycan attached on the serine residue of mucin protein is composed of N-Acetylgalactosamine, N-Acetylglucosamine, fucose, galactose and sialic acid and has similar structures to HMO. It has been reported that several intestinal bacteria are able to interact with the mucin glycans [25], [26]. Using mucin as the sole carbon source (Figure S1B), an increase in OD_600_ was appreciated for *B. infantis* albeit with the high initial cell density values.

The number of proteins identified from the soluble and insoluble fractions is summarized in Table S3. Between 350 and 400 proteins were found in any individual sample grown on each carbon source, and 100∼170 proteins were commonly observed in both the soluble and insoluble fractions in each sample. A sum total of 540 proteins were identified during growth on the seven different carbon sources among 2416 ORFs encoded by *B. infantis.*

Using the normalized spectrum abundance factor (NSAF) for each protein (representing the normalized protein quantity in a whole proteome of a single sample), quantitative expression profiles of whole proteomes were compared between cells grown on different carbon sources. While growth on most substrates exhibited similar proteome expression patterns, growth on galactooligosaccharides resulted in a separate cluster as witnessed by hierarchical cluster analysis (Figure S2). In addition, the correlation coefficients (Pearson’s product) between the GOS and the other proteomes were low (0.6∼0.8), indicating that the protein expression observed during growth on GOS is significantly different from growth on other tested substrates (Table S4).

### Validation of Cell Wall Associated (CWA) Proteomics

Most proteins observed in current shotgun whole cell proteomics experiments are cytosolic, while cell wall associated (CWA) proteins (both cell wall attached and membrane proteins) remain in the insoluble fraction with cell debris. Even if released from the soluble fraction, they are often missed due to the low ionization efficiency during MS analysis. Another constraint affecting their detection is their low (and/or variable) abundance by comparison to more readily obtained cytosolic proteins. To better understand the expression of CWA proteins, the insoluble fraction obtained after cell disruption was analyzed separately followed by the extensive washing with a strong denaturing agent (8 M urea/0.1M Tris). After removal of cytosolic proteins by washing, peptides from the CWA proteins were released by tryptic digestion. Protein localization was estimated by the quantitative ratio of their amounts in the soluble fraction and insoluble fraction.

**Figure 1:**
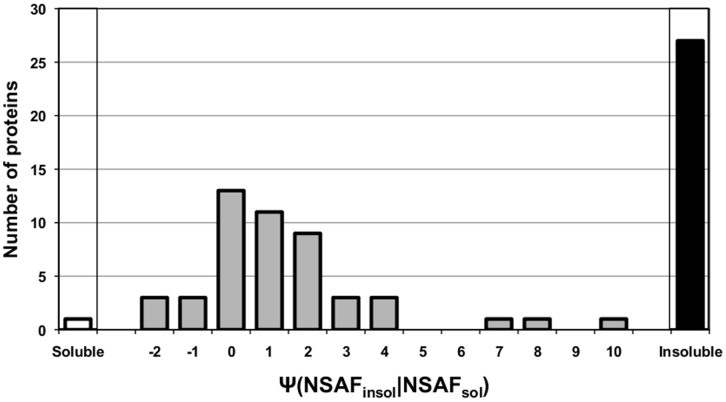
Distribution of the NSAF ratio of each SBP between soluble and insoluble fraction (grey bars; ψNSAF_insol_/NSAF_sol_). White bars (soluble: left corner) and black bars (insoluble: right corner) indicate the number of SBP observed solely in soluble and insoluble fraction, respectively.

To validate this approach, the expression of representative CWA proteins such as extracellular solute binding proteins (SBPs) from *B. infantis* ATCC 15697 was examined [27]. These are common lipoproteins often associated with the transport of various ligands. Overall, 71 incidences of SBP expression were observed from the proteome of *B. infantis* grown on the seven different carbon sources. Among these, 23 cases of SBP expression were only found in the insoluble fraction (Figure 1, black bar). Moreover, 93% of the observed SBPs exhibited in average 2.9-fold higher NSAF value in the insoluble fraction by comparison to the soluble fraction (Figure 1). In addition to the SBPs, expression of other known cell wall proteins was more clearly, or solely, witnessed in the insoluble fraction. As shown in Table S5, Blon_1259, a V5/Tpx-1 family protein was not identified on the soluble fraction during growth on any carbon source, but it was one of the most abundant proteins observed in insoluble fraction. Also a cell surface protein involved in capsular exopolysaccharide synthesis (Blon_2082) and the NlpA lipoprotein (Blon_1721) exhibited 2 and 14 fold higher NSAF values in the insoluble fractions than that observed in the soluble fractions respectively.

Therefore, by comparing the NSAF values of the soluble and insoluble protein fractions this approach provided insight into the cellular localization of each protein identified (Table 1 and 2). As shown on Table 1, 196 proteins were classified as CWA proteins and 93 were cytosolic (CYT) protein. However, the remaining 281 proteins could not be conclusively classified in either cellular location (UI: unidentified). To further evaluate our method, the number of a theoretical CWA protein was predicted bioinformatically [28]. Determination of transmembrane domains and signal peptides by TMHMM 2.0 [29] and the SignalP 2.0 HMM [30] indicated that 127 proteins are putative cell surface associated proteins. Among this pool of proteins, 108 were validated as cell wall associated by this proteomic approach. Only two of the cell surface associated proteins identified bioinformatically were categorized as cytosolic. In addition, 17 out of 127 theoretical surface proteins were found in the UI group. Thus while 281 proteins were classified into this group by the above scoring criteria, the latter results on putative cell surface proteins suggests that the UI group is more likely to contain cytosolic proteins.

**Table 1:** Distribution of *B. infantis* proteins in cell wall or cytosolic fractions.

Location	Proteins identified^a^	Theoretical cell wall/membrane associated proteins^b^
Cell wall association (CWA)	196	108
Cytosol (CYT)	93	2
Unidentified (UI)	281	17
Total	540	127

a Number of the protein for which the location was determined by the score system (See Materials and Methods).

b Theoretical cell wall/membrane associated proteins were determined by the Signal prediction by SignalP 2.0 HMM and the transmembrane helices prediction by TMHMM2.0. Bioinformatic information including pfam classification was obtained from the Integrated Microbial Genomes database.

### Carbohydrate Metabolism

**Figure 2:**
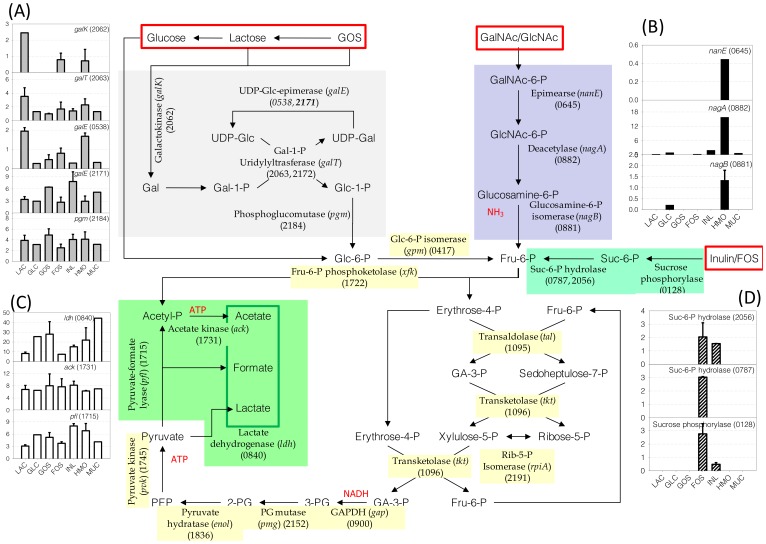
Central metabolic pathways reconstructed from proteomic datasets. Sugar substrates are presented inside the red boxes. Pathways regulated or induced by the presence of a specific sugar are indicated by colored background and their expressions on different sugars are presented next to the pathway as bar graphs: (a) Leloir pathway, (b) HexNAc catabolic pathway, (c) pyruvate fermentation pathway, (d) FOS/Inulin glycosyl hydrolase. The relative amounts of enzymes involved in the bifid shunt are presented in Figure S3. Numbers in the parenthesis next to the enzyme are the locus tag of each protein expressed.

Proteins involved in carbohydrate metabolism in *B. infantis* are summarized in Figure 2. Enzymes in the bifid shunt and glycolytic pathway, which are common to the utilization of various carbon sources, exhibited constitutive expression on the substrates used in this study (Figure S3). When compared to lactose-grown cells, the amount of each protein in these pathways varied less than two-fold among the different substrates. Interestingly, the genome of *B. infantis* ATCC 15697 indicates that several enzymes have isozymes. For example it contains seven genes encoding putative phosphoglycerate mutases, but only the expression of a single gene was appreciated, Blon_2152 (Figure S3).

*B. infantis* metabolizes galactose through the Leloir pathway. Proteins of this pathway were observed in the proteomes of cells grown on all seven carbon sources, albeit their amounts varied. As shown in Figure 2A, galactokinase (*galK*: Blon_2062), Gal-1-P uridylyltransferase (*galT*: Blon_2063) and UDP-glc epimerase (*galE*: Blon_0538) were highly expressed on lactose, HMO and FOS. However, the alternative *galE* (Blon_2171) and phosphoglucomutase (*pgm*: Blon_2184) were constitutively expressed across all substrates.

N-Acetylglucosamine (GlcNAc) is one of the major constituents of HMO. To metabolize this monosaccharide, *B. infantis* possibly converts GlcNAc to fructose-6-P (Fru-6-P) by deacetylation followed by deamidation. GalNAc is another hexosamine found in O-linked glycans, and it could be first isomerized to GlcNAc by an N-Acetylglucosamine 6-phosphate 2-epimerase (*nanE*: Blon_0645). The acetyl and amine groups of GlcNAc-6-P could then be removed by GlcNAc-6-P deacetylase (*nagA*: Blon_0882) and glucosamine-6-P isomerase (*nagB*: Blon_0881), respectively. These three enzymes are expressed significantly only when *B. infantis* was cultivated on HMO, suggesting the specific induction of catabolic pathway for HexNAc (Figure 2B).

While the proteins in the main glycolytic pathway were constitutively expressed, enzymes involved in pyruvate fermentation changed their expression level depending on the carbon source. As shown in Figure 2C, pyruvate is reduced to lactate by lactate dehydrogenase (*ldh*: Blon_0840) with the regeneration of NAD^+^. Alternatively, pyruvate is converted to formate and acetate by the combination of pyruvate-formate lyase (*pfl*: Blon_1715) cleaving pyruvate to formate and acetyl CoA, and acetate kinase (*ack*: Blon_1731) converting acetyl CoA to acetate with the production of ATP. Expression of acetate kinase and formate C-acetyltransferse was consistent across all growth substrates. However, lactate dehydrogenase exhibited a different expression pattern depending on the carbon sources used. During growth on lactose or FOS, the amounts of LDH were 2 to 3.5-fold smaller than those when *B. infantis* grew on glucose, GOS, inulin and HMO. Interestingly, LDH was expressed 5.5-fold more on mucin compared to lactose. Alternative route enzymes that can produce acetyl CoA from pyruvate, i.e. pyruvate dehydrogenase or pyruvate oxidase, were not expressed in any of the proteomes (data not shown).

Inulin and FOS are fructose polymers with different degrees of polymerization (DP). When FOS and inulin were used as carbon sources, a specific glycosyl hydrolase (GH) was induced (Figure 2D). Blon_0128 is a family 13 GH annotated as a sucrose phosphorylase. These enzymes cleave sucrose to glucose and fructose-6-phosphate. The NSAF of Blon_0128 in the soluble fraction is higher than insoluble fraction suggesting a cytosolic localization. Two family 32 GH proteins (Blon_2056 and Blon_0787) were also expressed exclusively on FOS and/or inulin. Known activities of the family 32 GH includes inulinase (EC:3.2.1.7), levanase (EC:3.2.1.65), and exo-inulinase (EC:3.2.1.80). While Blon_0128 and Blon_2056 were expressed on both inulin and FOS, the expression of Blon_0787 was observed only in the proteome of FOS grown cells. The localization of Blon_0787 was determined in the UI group but is most likely a cytosolic protein.

### Solute Binding Proteins (SBP)

**Figure 3:**
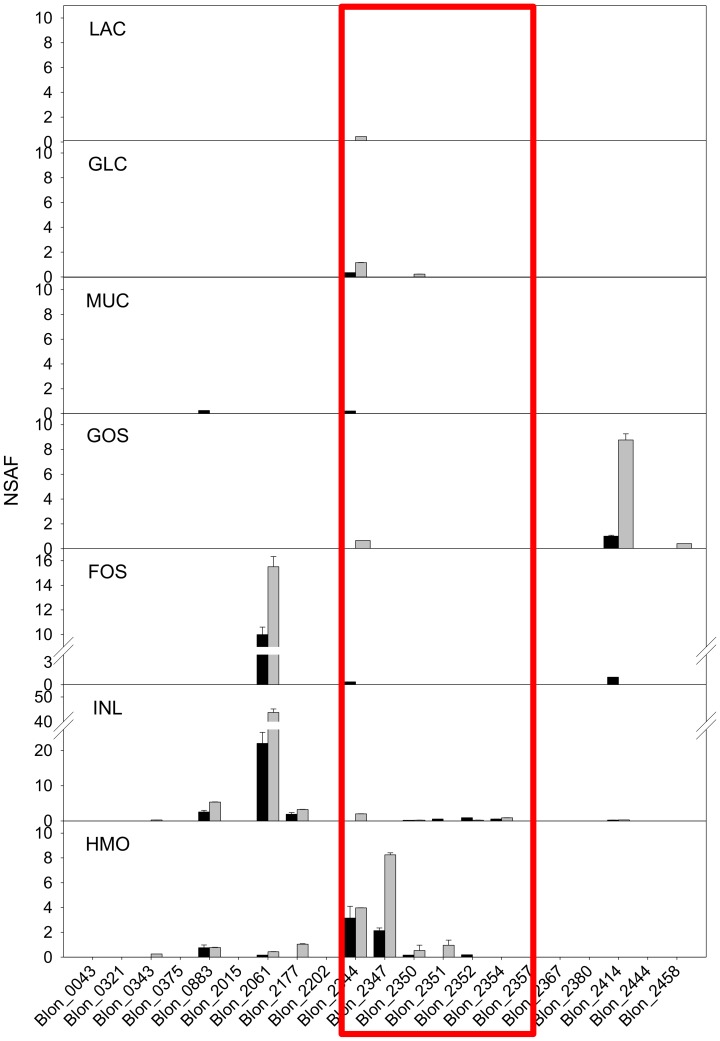
The expression of Family 1 extracellular SBPs on different carbon sources. The quantitative expression of each SBP is described by NSAF value. Black and gray bars indicate the NSAF values obtained in insoluble and soluble fraction, respectively. Box indicates the SBPs in the HMO cluster.

Extracellular SBPs are typically linked to specific ABC transport systems consisting also in two inner membrane transporter components and an ATPase. The *B. infantis* genome contains 39 SBPs of three different classes: Family 1, 3, and 5 [31]. In particular, Family 1 extracellular SBPs are thought to be responsible to the translocation of oligosaccharides [31]. As shown in Figure 3, different SBPs were expressed during growth of *B. infantis* on different carbohydrates. As indicated above, all Family 1 SBPs were categorized as CWA.

When *B. infantis* was grown on GOS, the amounts of SBP Blon_2414 were notably high, and FOS and inulin induced the expression of Blon_2061. In the latter case, low amounts of other SBPs were also observed, however, the expression of Blon_2061 was 10∼20 fold higher than the others. When HMO was added on the media, SBPs Blon_2344 and Blon_2347 were mainly expressed. Significant expression of SBPs was not observed when *B. infantis* grew on glucose and lactose. Interestingly, mucin, whose glycan structure is similar to HMO, did not induce the expression of any of Family 1 SBPs.

*B. infantis* has seven Family 3 and nine Family 5 SBPs, with broad affinity for di- and oligopeptides. Family 3 SBPs were actively expressed throughout cell growth (Figure S4). Blon_0747 and Blon_0760 were commonly expressed regardless of carbon sources. Blon_2021 was induced by glucose, lactose, GOS, FOS and mucin but not HMO or inulin. Expression of Blon_0710 and Blon_2022 was observed when *B. infantis* was cultivated on glucose and lactose. Among Family 5 SBPs, Blon_0922 was highly expressed on all carbon sources examined. The amount of Blon_0922 expression was consistent and very high: it was one of the most highly expressed SBPs on all conditions (with the exception of Blon_2061 during growth on inulin). NSAF of Blon_0834 was high but specific in GOS proteome. Blon_0053 and Blon_2419 appeared to be expressed constitutively across all substrates but at a low level.

### HMO Cluster I Protein Expression

**Figure 4:**
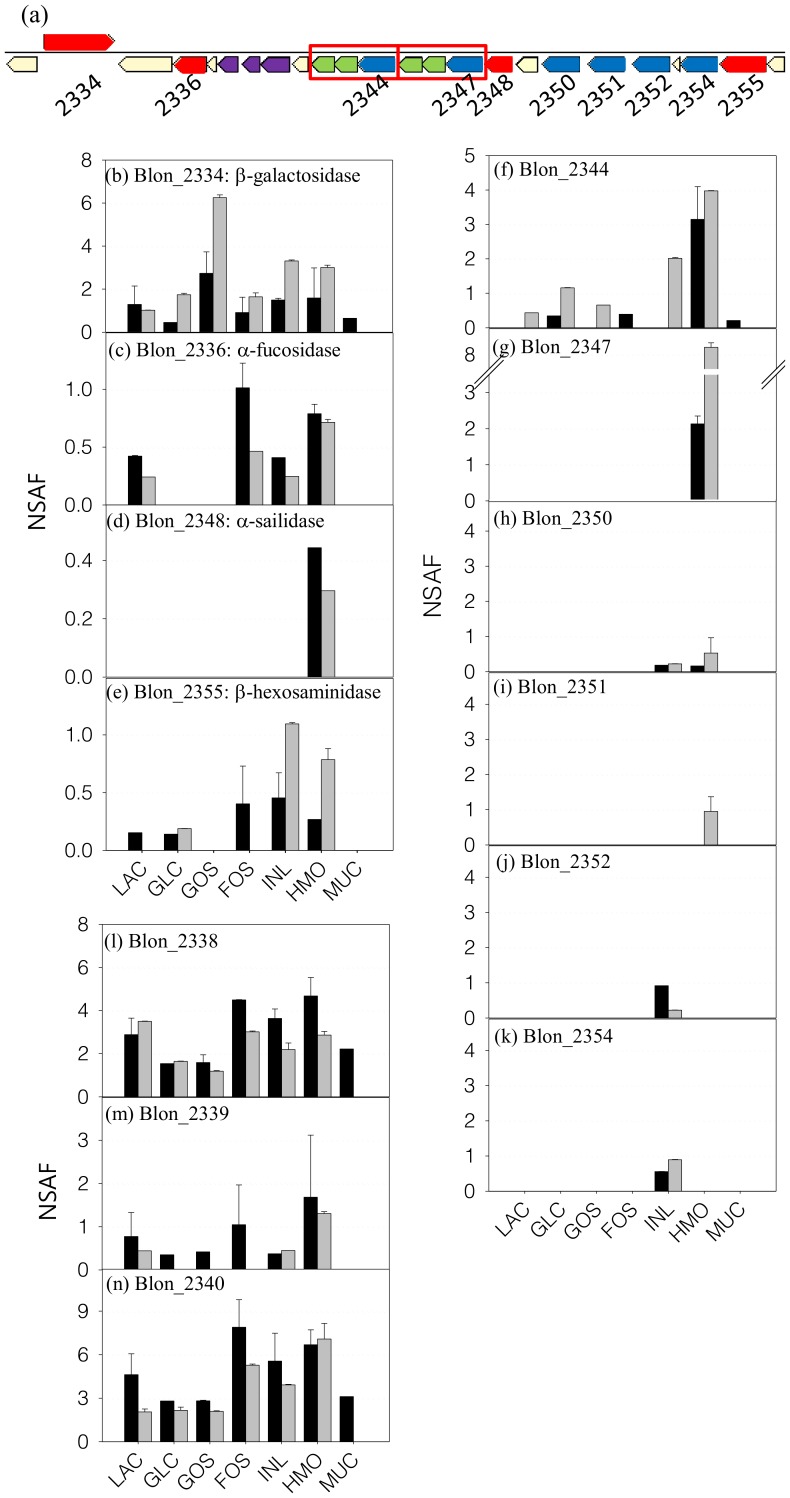
The expression of proteins in HMO cluster in *B. infantis* during growth on different prebiotics. (a) Graphical display of genes in HMO cluster. Proteins involved in the HMO translocation and degradation are noted with the locus tag under the gene. Sets of genes inside the red box are the transport systems that comprise a Family 1 extracellular SBP and two inner-membrane components. Bars represent the relative amounts of each protein in different carbon sources. Black and gray bars indicate the NSAF values in the soluble and insoluble fraction, respectively. (b)∼(e) are the glycosyl hydrolases for HMO degradation. (f)∼(k) are the Family 1 SBPs and (l)∼(n) are the proteins whose the amount was high but the role in HMO metabolism is not well known.

*B. infantis* contains a 43 kb cluster (HMO cluster I) that encodes genes for the catabolism of HMO ([11]; Figure 4A). During growth on HMO, expression of all different glycosyl hydrolases required for the degradation of HMO was observed. A β-galactosidase (Blon_2334) was expressed on all seven proteomes but the expression during growth on GOS was 2∼3 times higher than that of other carbon sources (Figure 4B). The expression of the α-1/3,4 fucosidase Blon_2336 [32] was observed on lactose, FOS, inulin, and HMO indicating a more constitutive expression (Figure 4C). Interestingly, the expression of an α-sialidase Blon_2348 [33] was exclusive to the HMO proteome (Figure 4D). The expression of a β-hexosaminidase (Blon_2355) was observed only during growth on HMO and inulin (Figure 4E). Using the scoring criteria described above, the cellular location of β-galactosidase and β-hexosaminidase were inconclusive however the NSAF of these proteins in the insoluble fraction was more than 2 times higher than those at soluble fraction suggesting possible membrane association. As described in Figure 4A, putative HMO-related transporter SBPs Blon_2344 and Blon_2347 were adjacent to genes for two inner membrane transporter components, while SBPs Blon_2350, Blon_2351, Blon_2352, and Blon_2354 were positioned elsewhere in the HMO Cluster 1 without adjacent permease components. Protein from Blon_2344 was identified during growth on most of carbon sources but its amount was low (Figure 4F). During growth on HMO, however, expression of Blon_2344 was increased significantly. Blon_2347 was only expressed when cultivated on HMO (Figure 4G). Other Family 1 SBPs in the HMO Cluster 1 were determined (if detected at all) in small amounts, suggesting a basal level expression (Figure 4H-K). Proteins Blon_2338, Blon_2339, and Blon_2340 are also located on the HMO cluster 1, and they were expressed constitutively (Figure 4L–N). Their localization appears to be cytosolic however their function and/or relationship to HMO utilization are unclear.

### Glycosyl Hydrolases (GH) and Glycosyltransferases [34]

**Figure 5:**
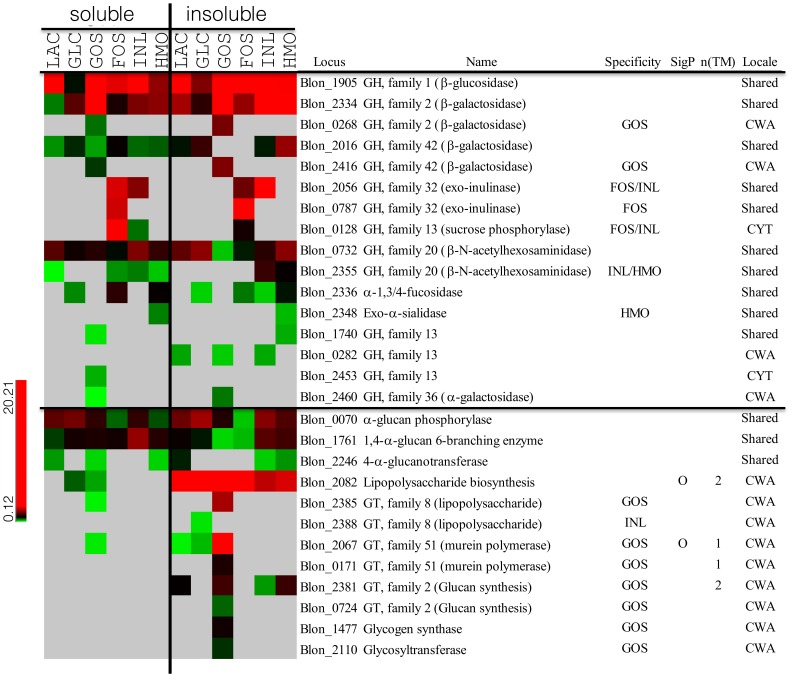
The expression of glycosyl hydrolases and glycosyltransferases in *B. infantis* during growth on different prebiotics. The amount of protein expressed scaled by color from green to red as shown in the legend. Gray indicates that the expression was not detected. Left and right panel of heat map represent the expression of protein in soluble and insoluble fraction, respectively. CWA protein was determined by the NSAF ratio between soluble and insoluble faction (NSAF_insol_/NSAF_sol_≥2.5).

Glycosyl hydrolases (GHs) and glycosyltransferases (GTs) are important enzymes that degrade or synthesize oligosaccharides respectively. Expression of 16 GHs was observed throughout the proteomes obtained (Figure 5). Blon_1905 is Family 1 GH, presumably a β-glucosidase, which was constitutively and highly expressed during growth on all of the different carbon sources. Expressions of four different β-galactosidases were identified. Blon_2334 (GH Family 2) and Blon_2016 (GH Family 42) were constitutively and highly expressed on all substrates, in order with previous observations [35]. Blon_0268 (GH2) and Blon_2416 (GH42) were specifically found during growth on GOS. Interestingly, NSAFs of Blon_2334, Blon_0268, and Blon_2016 in the insoluble fraction were two-fold higher than those in soluble fraction, suggesting a cell wall association of these GH activities. Three other GHs (Blon_2056, Blon_0787 and Blon_0128) were specifically expressed when *B. infantis* was grown on FOS and inulin. Blon_2056 and Blon_0787 are GH Family 32 proteins, suggested to be exo-inulinases [36]. The cellular location of these two enzymes was not conclusively determined. Blon_0128 is a GH Family 13 protein, annotated as sucrose phosphorylase, and was classified here as a cytosolic protein. In addition to Blon_2355 in the HMO cluster I, *B. infantis* has a second β- hexosaminidase (Blon_0732; GH20). While Blon_2355 was specifically expressed during growth on HMO and inulin and was cell wall-associated, Blon_0732 was constitutively expressed and is more likely cytosolic showing a similar or lower NSAF in the insoluble fraction. Finally, the expression of Family 13 (Blon_2456) and Family 36 (Blon_2460) GHs were found in GOS proteome however their amounts were relatively low compare to other GHs.

Enzymes involved in the glycan synthesis and exopolysaccharide production (Figure 5) were also identified. Blon_2246 and Blon_1761 are 4-α-glucanotransferase and 1,4-α-glucan 6-branching enzymes respectively, and both were consistently expressed regardless of the carbon source. An α-glucanphosphorylase (Blon_0070, GT family 35) which reversibly produces glucose-1-P from glucan was also constitutively expressed on all substrates. The NSAF ratio between soluble and insoluble fraction is close to 1.0-fold or less suggesting intracellular localization. Nine glycosyl transferases and one extracellular polysaccharide synthesis protein were identified and found to be highly enriched in the insoluble fraction suggesting a cell surface association. Among them, Blon_2082 is predicted to participate in the synthesis of exopolysaccharide, and it was found to be present in all of the proteomes analyzed regardless of the growth substrate. With the exception of Blon_2381, other CWA glycosyl transferases were expressed only during growth on GOS (Figure 5).

### Hypothetical Proteins

There are 845 hypothetical or unknown proteins in the *B. infantis* ATCC 15697 genome, which represent 35% of total ORFs [11]. Among these proteins, we observed expression of 63 proteins across seven different sugars (Figure S5). Most were cytosolic proteins expressed without significant variation in abundance among the growth substrates used. Remarkably, some of the cytosolic hypothetical proteins were highly expressed as high as glyceraldehyde-3-P dehydrogenase.

A notable result was witnessed regarding the expression of hypothetical CWA proteins. When *B. infantis* was grown on GOS, hypothetical CWA proteins were highly induced and/or significantly overexpressed. 26 hypothetical CWA proteins were observed from the proteome during growth on GOS while 8 to 17 hypothetical CWA proteins were found in the proteomes during growth on the other substrates, Furthermore, the expressed amount of these 26 CWA proteins observed on the GOS proteome was significantly higher than those from other substrates (Figure S5).

## Discussion

Mass spectrometry analysis of whole cell proteins is a common method to directly obtain information on the existing proteins within a biological system thus enabling the prediction of active metabolic pathways or regulatory networks. Because proteins have a slower turnover rate than mRNA and metabolites, proteomics can provide a more stable snapshot of cell metabolic system at the moment when cells were collected. While the cytosolic proteins are readily analyzed quantitatively and qualitatively, analysis of CWA proteins is still challenging due to the difficulties in fractionation and purification. “Shaving” the surface exposed proteins of intact cells is promising particularly in high-throughput analysis of whole cell proteome [19], [28], [37]. However, sequestering of cytosolic proteins into the insoluble fraction is often observed and it biases cell surface proteomics significantly [38], [39], [40]. Indeed, when we applied the shaving reaction to the intact cell of *B. infantis*, the proteomic results were the same as those obtained from cytosolic fraction (data not shown).

To improve the determination of the CWA proteome, two approaches were employed for *B. infantis*. Instead of intact cells, we disrupted *B. infantis* cells first and the remaining cytosolic proteins, as well as cellular exopolysaccharide, were eliminated with extensive washes using a strong denaturing buffer (8M urea/0.1M Tris). Then, the CWA proteins were cleaved with trypsin from the cell debris obtained from the insoluble fraction. Despite the enhanced number of cell wall proteins observed using this approach, highly expressed cytosolic proteins (i.e. enolase or pyruvate kinase) were still found as major proteins in the insoluble fraction (Table S6). Therefore we ascribed cell wall association by the relative ratio of protein abundance between the soluble and insoluble fractions. As shown in Figure 1, most of the extracellular SBPs exhibited more than a three-fold increase in protein abundance in the insoluble fraction and 23 SBPs were observed only in insoluble fraction. Meanwhile the cytosolic proteins exhibited the similar or less relative abundance in the insoluble fraction. For example, the relative abundance of enolase and pyruvate kinase was 1.55±0.43 and −1.14±0.29, respectively, suggesting these enzymes should not be classified as CWA proteins.

The determination of protein localization using the NSAF ratio of proteins observed in soluble and insoluble fractions was validated by comparison with the bioinformatic properties of the target proteins. Among the 127 proteins identified that possessed transmembrane domains or signal peptide sequences, 108 of them were determined as CWA proteins. Only two theoretical cell wall/membrane proteins were determined exclusively as cytosolic (Table 1). Of the 196 proteins that were classified as CWA proteins, 88 proteins did not present any bioinformatic evidence for the cell wall/membrane association. However, cell wall association defined here is not necessarily the indication of protein integration into the cell membrane or extracellular position *per se*. Since the tryptic digestion is performed after the disruption of cell, proteins associated with the inner part of cell membrane may also be captured.

**Figure 6:**
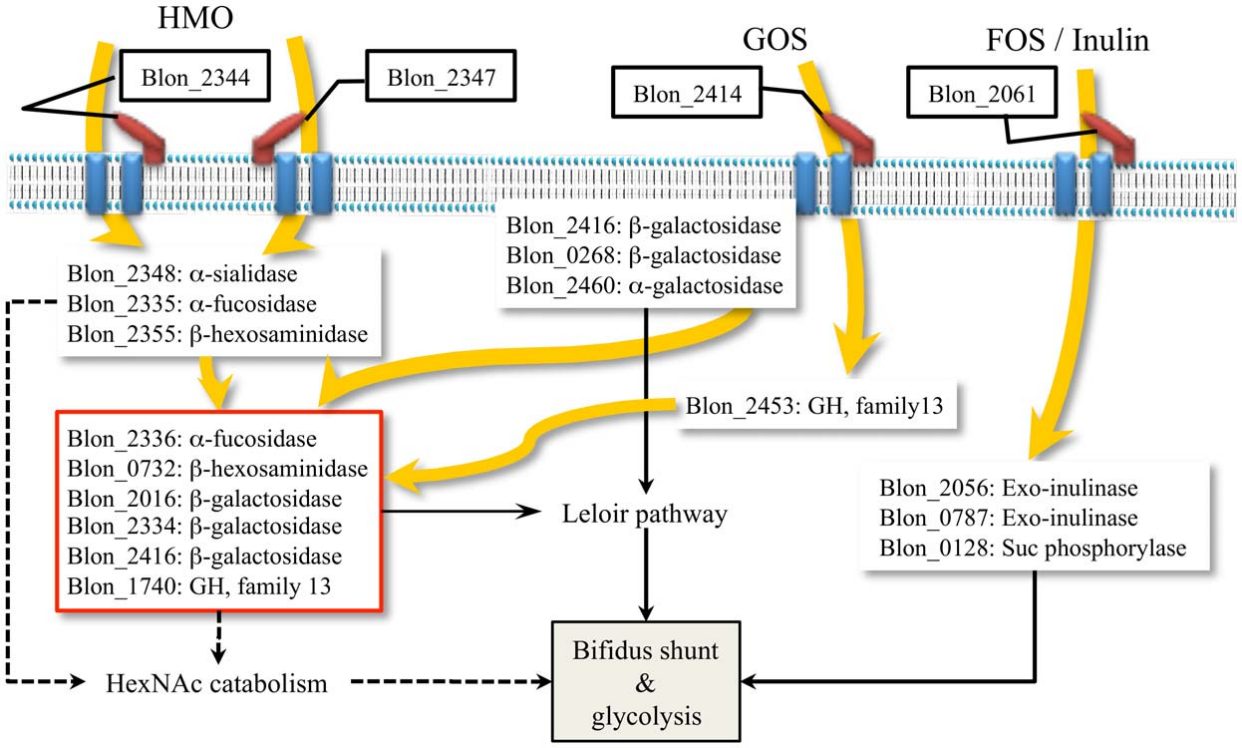
Schematic diagram of the potential metabolic pathways for different prebiotic consumption in *B. infantis*. The catabolic pathways for monosaccharides were detailed in Figure 2. Bold and curved arrows indicate the flow of oligosaccharide.

It has been shown that *B. infantis* can utilize various prebiotics [27], [41], [42], [43]. However, the cognate pathways that metabolize these substrates have not been investigated. Whole cell proteomics enabled us to determine the expression of key cellular proteins involved in the catabolic process for different prebiotics (Figure 6). HMO, GOS, FOS and inulin are oligosaccharides with different chemistries and degrees of polymerization. In particular, HMO is a heterologous mixture of more than 200 different structural and compositional isomers [5]. In *B. infantis*, complete hydrolysis of HMO to monosaccharides appears to occur in the cytosol after translocation [9]. As shown in Figure 3, induction of specific Family 1 SBPs during growth on different oligosaccharides suggests these are transported inside the cells by associated ABC transport systems. Family 1 SBPs are induced during growth on HMO bind different isomers of these molecules and we previously observed a similar SBP expression pattern via proteomic analysis and transcriptomic analysis [27]. In addition, two Family 1 SBPs, a β-hexosaminidase, an α-fucosidase and an α-sialidase from the main HMO cluster were expressed only when *B. infantis* grew on HMO as the sole carbon source. Proteomics also suggested that both inulin and FOS are imported by the transporter associated to the Family 1 SBP Blon_2061, which are then cleaved by two exo-inulinases (Blon_2056 & Blon_0787), with the help of a sucrose phosphorylase (Blon_0128) to degrade the polymer to hexose phosphate for further metabolism. GOS appears to have specific transport and hydrolysis system. Blon_2414 was the only Family 1 SBPs expressed during growth on GOS, and the adjacent β-galactosidase (Blon_2416) was also induced by GOS.

*B. infantis* contains both constitutively expressed and inducible glycosyl hydrolases (Figure 6). While a set of GHs was expressed in association with specific oligosaccharides, β-galactosidases Blon_2334 and 2016, β-hexosaminidase Blon_0732 and an oligosaccharide hydrolase (Family 13 GH: Blon_1740) were to a different extent stably expressed in all carbon sources. The α1-3/4 fucosidase Blon_2336 was highly expressed during growth on HMO. The activity of several of these enzymes on HMO has been recently explored [32], [33], [35], [44]. A series of β-galactosidases, as well as Family 13 GHs, were expressed when *B. infantis* was grown on GOS (Figure 5). Most of the GHs identified were determined to be cytosolic (Figure 5). However, two β-galactosidases (Blon_0268 and Blon_2416) and one α-galactosidase (Blon_2460), which were expressed only grown on GOS, were classified as CWA proteins. The lack of transmembrane domain or signal peptide sequence might imply the proximity to the cell wall/membrane inside the cytosol instead of extracellular display.

After hydrolysis of oligosaccharides, the constituent monosaccharides or their phosphorylated forms are further metabolized through common central pathways. Glucose and fructose directly enter the bifidus shunt, for which the cognate enzymes appear to be constitutively expressed (Figure 2). Galactose enters the central carbon metabolic pathways via the Leloir pathway. The enzymes in this pathway were constitutively expressed in *B. infantis*, but interestingly GalK and GalE were up-regulated in the presence of sugars that contain galactose (i.e. lactose, GOS or HMO). Enzymes that convert GlcNAc to fructose-6-P were only expressed when HMO was in the media suggesting their induction by GlcNAc.

Mucin is a highly polymerized protein that contains O-linked glycans at specific serine or threonine residues. Because mucin O-glycans are structurally similar to HMO, it is likely a similar set of enzymes metabolize these substrates in bifidobacteria. However, growth on mucin by *B. infantis* did not lead to a similar proteomic profile compared to growth on HMO, suggesting no hydrolysis or metabolism of these O-glycans. In turn *B. infantis* is able to cleave and consume N-linked glycans [45]. In contrast, *Bifidobacterium bifidum* PRL2010 can metabolize both HMO and mucin [26] by similar mechanisms including membrane glycosyl hydrolases and permeases [26], as previously determined by proteomics and microarrays. Thus *B. bifidum* and *B. infantis* represent convergent strategies for HMO consumption in the infant gut microbiota [9].

The main end products of bifidobacterial metabolism are usually lactic acid, acetate and formate [11]. Acetate kinase (*ack*) converts acetyl-P to acetate and ATP (Figure 2C). This enzyme was constitutively expressed across the growth substrates used. Conversely, lactate dehydrogenase (*ldh*), producing lactate from pyruvate with the conversion of NADH to NAD+, was differentially expressed depending on the carbon source. Interestingly, this enzyme was present at lower levels during growth on FOS and inulin, and this correlates with higher acetate:lactate ratios during growth on these prebiotics [46]. Production of certain short chain fatty acids by commensal bacteria has been shown to have protective effects against pathogens [47]. The higher amount of *ldh* protein observed for certain growth substrates such as GOS and HMO implies higher levels of lactate as an end-product.

It has been reported that species of *Bifidobacterium*, including *B. infantis*, are able to produce exopolysaccharides (EPS) and their structures have been elucidated [48], [49], [50], [51], [52], [53], [54]. Indeed, Tone-Shimokawa *et al* revealed that the EPS produced by the *B. infantis* ATCC 15697 (formally referred as *B. infantis* Reuteri ATCC 15697) is a galactose polymer with the structure of β-D-Gal-(1→3)-α-D-Gal [55]. Important functions in host-microbe interactions such as stress tolerance and immunomodulation have been recently determined for *B. breve* EPS [56]. In addition, bifidobacterial genomes indicate they can synthesize storage polysaccharides such as glucan or glycogen [11]. Three enzymes in *B. infantis*, α-glucan phosphorylase, 1,4-α-glucan 6-branching enzyme, and 4-α-glucanotransferase that might synthesize (or reversibly degrade) α-glucans were constitutively expressed (Figure 5). Interestingly, their expression was classified as cytosolic while other glycosyl transferases [34] were found associated the CWA fraction. Blon_2082, a lipopolysaccharide biosynthesis protein was highly and constitutively expressed on cell surface regardless of sugar. Expressions of the additional nine GTs were identified in the insoluble fraction. Eight of them, which included more than three different families of GT, were exclusively or highly expressed in the presence of GOS in the medium.

In conclusion, this study provides a global picture of the consumption of important prebiotics such as HMO, GOS and FOS by *B. infantis* ATCC 15697 with a focus on the proteins deployed for catabolism of these sugars. Using an improved proteomics approach to specifically determine cell-wall associated from cytosolic proteins, we have shown that in general utilization of these substrates occurs via specific transport mechanisms, defined by specific solute binding proteins, and glycosyl hydrolases that convert these oligosaccharides into monosaccharides. While several known proteins in the HMO cluster I were only expressed during bacterial growth on these substrates, GOS responses included an important number of hypothetical proteins, as well as individual β-galactosidases and SBPs.

## Supporting Information

Figure S1 **Cell growth profile of** ***B. infantis*** **ATCC 15697 grown on different carbon sources.** (A) *B. infantis* was cultivated in 15 ml of ZMB media and 2% (w/v) of carbon sources. Optical density (OD) was measured automatically at 600 nm without dilution. Carbon sources and their symbol were noted in the legend. LAC; lactose, GLC; glucose, FOS; fructooligosaccharide, INL; inulin, HMO; human milk oligosaccharide, GOS; galactooligosaccharides. (B) *B. infantis* was cultivated in 25 ml of M17 media with 2% (w/v) of glucose, mucin and HMO as a carbon source. Optical density of cell was measured with appropriate dilution within the range of 0.3∼0.5 at the wavelength of 600 nm.
(PDF)

Figure S2 **Hierarchical cluster analysis of protein expression profile of** ***B. infantis*** **grown on different carbon sources.** Similarity was calculated by Euclidian distance and the cluster was built by the Average linkage method. LAC, lactose; GLC, glucose; FOS, fructooligosaccharides; INL, inulin; HMO, human milk oligosaccharides; MUC, mucin; GOS, galactooligosaccharides.
(PDF)

Figure S3 **Expression of proteins involved in the glycolysis and the bifid shunt.** The comparative proteomics using the lactose proteome as control suggested that the expression level was changed less than two fold showing constitutive expression regardless of carbon sources. LAC, lactose; GLC, glucose; FOS, fructooligosaccharides; INL, inulin; HMO, human milk oligosaccharides; MUC, mucin; GOS, galactooligosaccharides.
(PDF)

Figure S4 Expression of (a) family 3 and (b) family 5 extracellular solute binding (SBP) proteins. Gray and block bars represent the NSAF values obtained from the proteome of the cell wall associated and the cytosolic fraction, respectively. LAC, lactose; GLC, glucose; FOS, fructooligosaccharides; INL, inulin; HMO, human milk oligosaccharides; MUC, mucin; GOS, galactooligosaccharides.
(PDF)

Figure S5 **Expression of hypothetical proteins.** Soluble and insoluble indicate the fractions where the proteins are located: cell wall associated (CWA), cytosolic (CYT), or shared, as described in Methods and materials. SigP and TM stand for the presence of signal peptide sequence and the number of transmembrane domains, respectively. LAC, lactose; GLC, glucose; FOS, fructooligosaccharides; INL, inulin; HMO, human milk oligosaccharides; MUC, mucin; GOS, galactooligosaccharides.
(PDF)

Table S1 The score system to determine the protein localization.
(PDF)

Table S2 The range of a score that determine the protein location.
(PDF)

Table S3 Number of proteins expressed in the *B. infantis* proteome across different prebiotics.
(PDF)

Table S4 Linear correlation coefficients (Pearson’s Product) between two proteomes.
(PDF)

Table S5 Normalized protein amounts of representative cell surface associated proteins in soluble and insoluble fraction.
(PDF)

Table S6 List of top 30 most expressed proteins in (a) soluble and (b) insoluble fractions. The rank was determined by the sum of all NSAF values of each carbon sources. The CWA proteins were marked by gray area. Ribosomal proteins were excluded in the list. LAC; lactose, GLC; glucose, GOS; galactooligosaccharides, FOS; fructooligosaccharides, INL; inulin, HMO; human milk oligosaccharides, MUC; mucin.
(PDF)
