# Supplementary material for: Proteomic Analysis of Bifidobacterium longum subsp. infantis Reveals the Metabolic Insight on Consumption of Prebiotics and Host Glycans
Source: PLoS One. 2013 Feb 26;8(2):e57535. doi: 10.1371/journal.pone.0057535 (PMC3582569; doi:10.1371/journal.pone.0057535)

Supplementary Figure S1 Cell growth profile of *B. infantis* ATCC 15697 grown on different carbon sources. (A) *B. infantis* was cultivated in the microplate reader with 15mL of DMZ media and 2% (w/v) of carbon sources. Optical density (OD) was measured automatically at 600 nm without dilution. Carbon sources and their symbol were noted in the onset. LAC; lactose, GLC; glucose, FOS; fructooligosaccharide, INL; inulin, HMO; human milk oligosaccharide, GOS; galactooligosaccharide (B) *B. infantis* was cultivated in 25mL of M17 media with 2% (w/v) of glucose, mucin and HMO as a carbon source. Optical density of cell was measured with appropriate dilution within the rage of 0.3~0.5 at the wavelength of 600 nm.

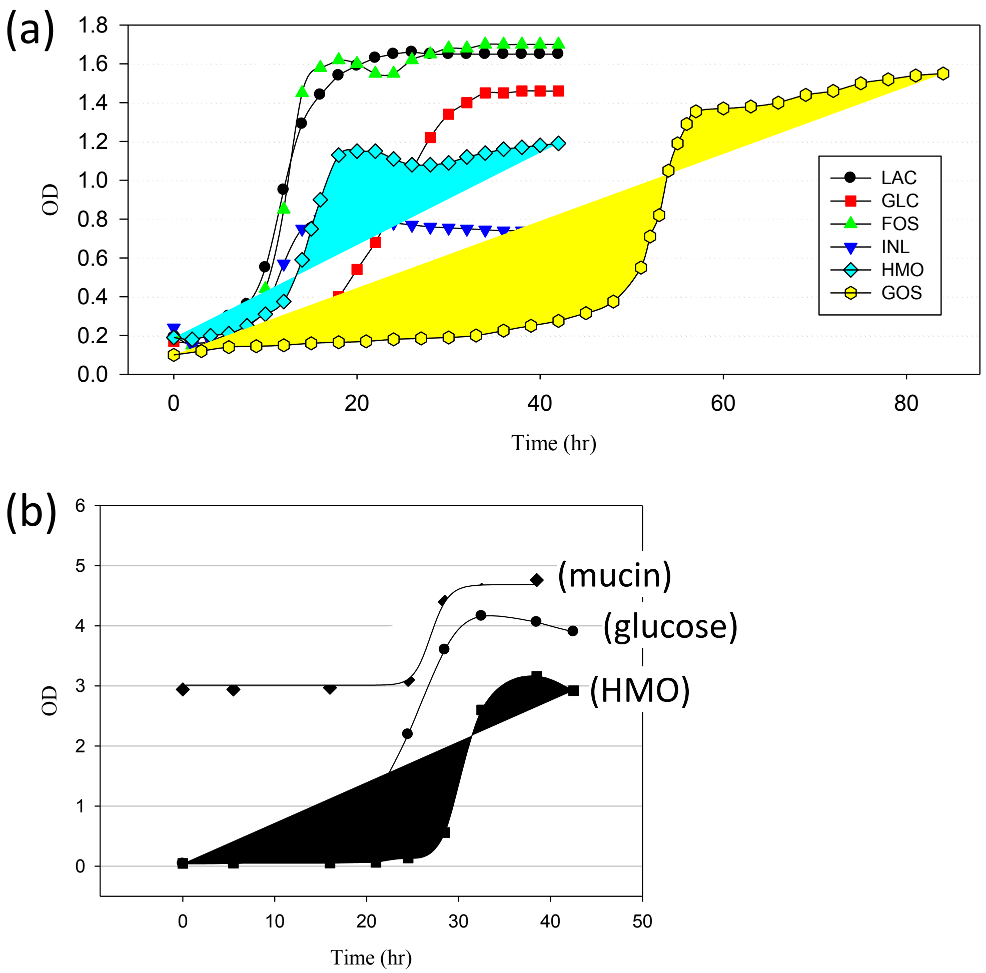

Supplement: Figure S1 — Cell growth profile of B. infantis ATCC 15697 grown on different carbon sources. (A) B. infantis was cultivated in 15 ml of ZMB media and 2% (w/v) of carbon sources. Optical density (OD) was measured automatically at 600 nm without dilution. Carbon sources and their symbol were noted in the legend. LAC; lactose, GLC; glucose, FOS; fructooligosaccharide, INL; inulin, HMO; human milk oligosaccharide, GOS; galactooligosaccharides. (B) B. infantis was cultivated in 25 ml of M17 media with 2% (w/v) of glucose, mucin and HMO as a carbon source. Optical density of cell was measured with appropriate dilution within the range of 0.3∼0.5 at the wavelength of 600 nm. (PDF) [file pone.0057535.s001.pdf]
