# Supplementary material for: Proteomic Analysis of Bifidobacterium longum subsp. infantis Reveals the Metabolic Insight on Consumption of Prebiotics and Host Glycans
Source: PLoS One. 2013 Feb 26;8(2):e57535. doi: 10.1371/journal.pone.0057535 (PMC3582569; doi:10.1371/journal.pone.0057535)

Supplementary Figure S2 Hierarchical cluster analysis of protein expression profile grown on different carbon sources. Similarity was calculated by Euclidian distance and the cluster was built by the Average linkage method. LAC; lactose, GLC; glucose, FOS; fructooligosaccharide, INL; inulin, HMO; human milk oligosaccharide, GOS; galactooligosaccharide

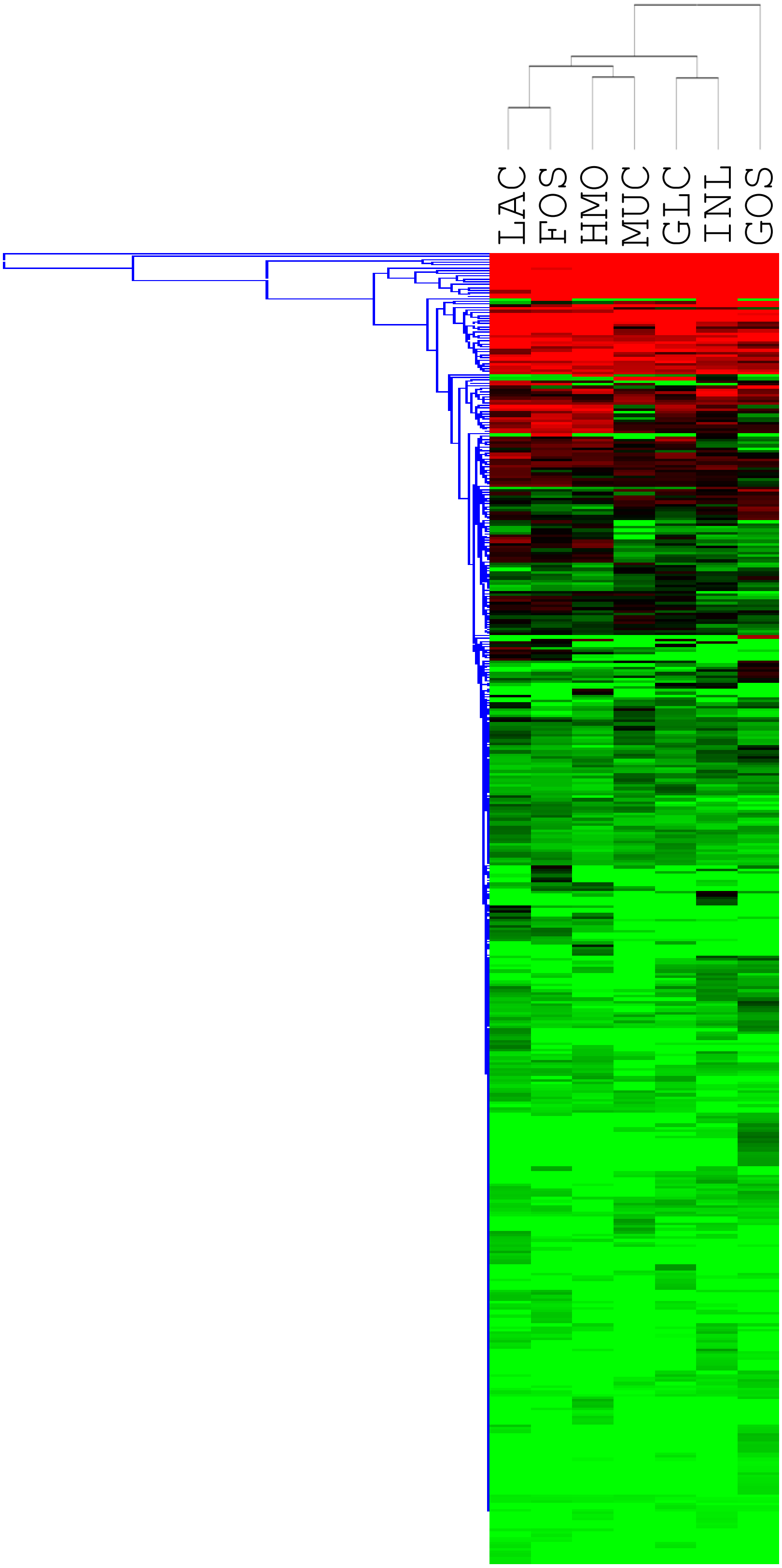

Supplement: Figure S2 — Hierarchical cluster analysis of protein expression profile of B. infantis grown on different carbon sources. Similarity was calculated by Euclidian distance and the cluster was built by the Average linkage method. LAC, lactose; GLC, glucose; FOS, fructooligosaccharides; INL, inulin; HMO, human milk oligosaccharides; MUC, mucin; GOS, galactooligosaccharides. (PDF) [file pone.0057535.s002.pdf]
