# Supplementary material for: Proteomic Analysis of Bifidobacterium longum subsp. infantis Reveals the Metabolic Insight on Consumption of Prebiotics and Host Glycans
Source: PLoS One. 2013 Feb 26;8(2):e57535. doi: 10.1371/journal.pone.0057535 (PMC3582569; doi:10.1371/journal.pone.0057535)

Supplementary Figure S3 Expression of proteins involved in the glycolysis and the bifidus shunt. The comparative proteomics using the lactose proteome as control suggested that the expression level was changed less than two fold showing constitutive expression regardless of carbon sources. LAC; lactose, GLC; glucose, FOS; fructooligosaccharide, INL; inulin, HMO; human milk oligosaccharide, GOS; galactooligosaccharide

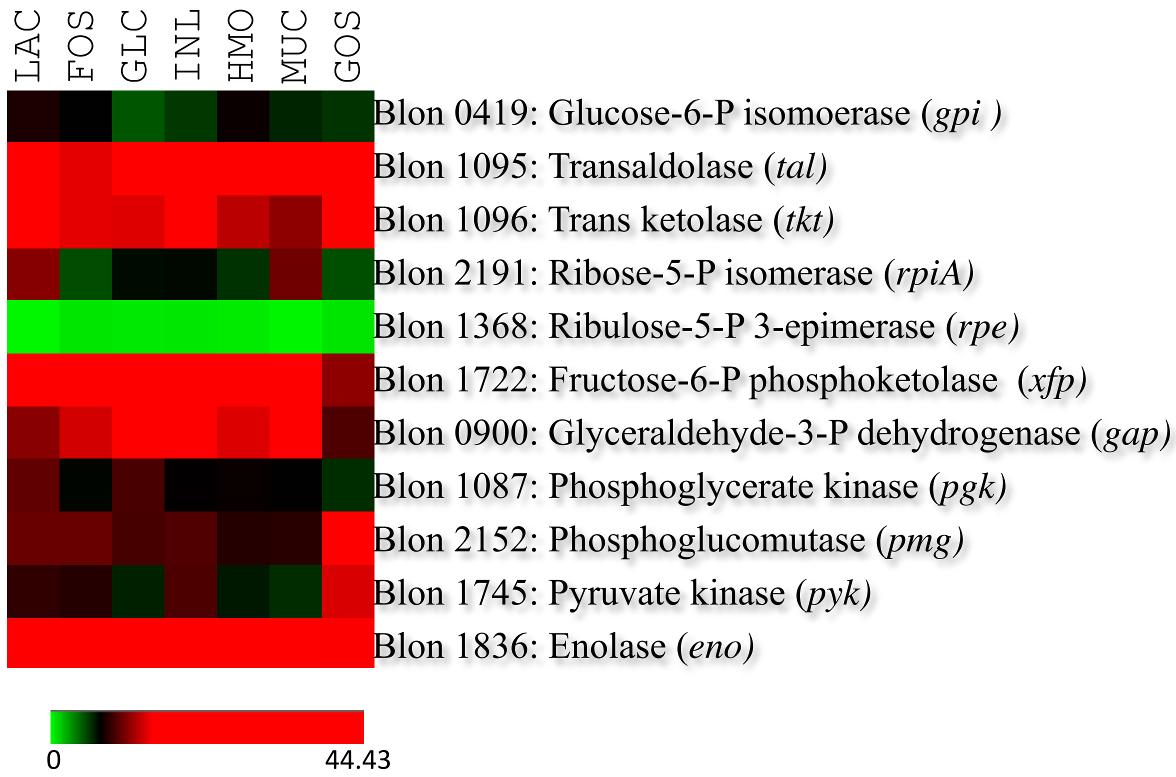

Supplement: Figure S3 — Expression of proteins involved in the glycolysis and the bifid shunt. The comparative proteomics using the lactose proteome as control suggested that the expression level was changed less than two fold showing constitutive expression regardless of carbon sources. LAC, lactose; GLC, glucose; FOS, fructooligosaccharides; INL, inulin; HMO, human milk oligosaccharides; MUC, mucin; GOS, galactooligosaccharides. (PDF) [file pone.0057535.s003.pdf]
