# Supplementary material for: Proteomic Analysis of Bifidobacterium longum subsp. infantis Reveals the Metabolic Insight on Consumption of Prebiotics and Host Glycans
Source: PLoS One. 2013 Feb 26;8(2):e57535. doi: 10.1371/journal.pone.0057535 (PMC3582569; doi:10.1371/journal.pone.0057535)

Supplementary Figure S5 Expression of hypothetical proteins. Soluble and insoluble indicate thee fractions that the protein isolated. CWA, CYT, Shared represent the protein location determined by the method explained in the manuscript (see Methods and materials).. LAC; lactose, GLC; glucose, FOS; fructooligosaccharide, INL; inulin, HMO; human milk oligosaccharide, GOS; galactooligosaccharide

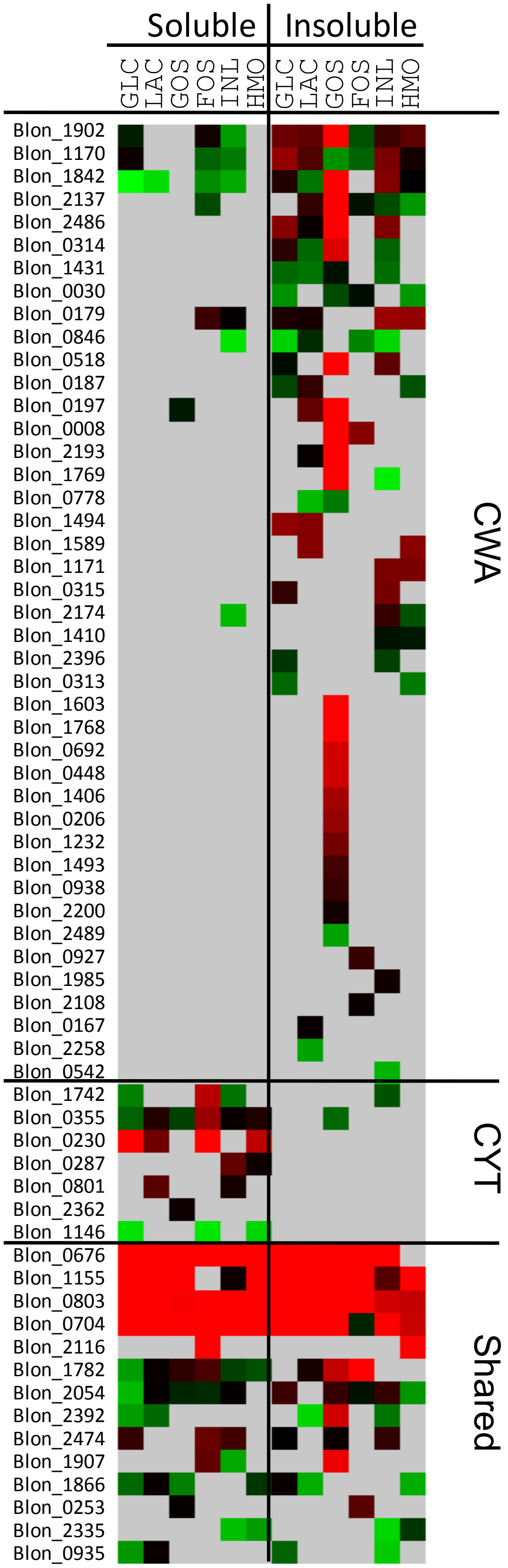

Supplement: Figure S5 — Expression of hypothetical proteins. Soluble and insoluble indicate the fractions where the proteins are located: cell wall associated (CWA), cytosolic (CYT), or shared, as described in Methods and materials. SigP and TM stand for the presence of signal peptide sequence and the number of transmembrane domains, respectively. LAC, lactose; GLC, glucose; FOS, fructooligosaccharides; INL, inulin; HMO, human milk oligosaccharides; MUC, mucin; GOS, galactooligosaccharides. (PDF) [file pone.0057535.s005.pdf]
