# Supplementary material for: Proteomic Analysis of Bifidobacterium longum subsp. infantis Reveals the Metabolic Insight on Consumption of Prebiotics and Host Glycans
Source: PLoS One. 2013 Feb 26;8(2):e57535. doi: 10.1371/journal.pone.0057535 (PMC3582569; doi:10.1371/journal.pone.0057535)
Supplement: Table S1 — The score system to determine the protein localization. (PDF) [file pone.0057535.s006.pdf]

Table S1: The score system to determine the protein localization.

Condition	Point
ID(CW)[a] *or* $\Psi(NSAF_{insol}\|NSAF_{sol})$[b] $\geq +3$	+1
ID(WCL)[a] *or* $\Psi(NSAF_{insol}\|NSAF_{sol}) \leq -3$	-1
$+2 < \Psi(NSAF_{insol}\|NSAF_{sol}) < +3$	+0.5
$-3 < \Psi(NSAF_{insol}\|NSAF_{sol}) < -2$	-0.5
$-2 < \Psi(NSAF_{insol}\|NSAF_{sol}) < +2$	0

[a]ID(CW), ID(WCL): Identified only at cell wall (CW) or whole cell lysate (WCL) fraction, respectively

[b] The NSAF ratio of a protein between CW and WCL fraction. If the NSAF at CW fraction was higher than the WCL fraction, the ratio had a positive value. Conversely, the ratio had a negative value. It can be calculated as follows.

,

$$\Psi(NSAF_{insol}|NSAF_{sol}) = \begin{cases} \dfrac{NSAF_{insol}}{NSAF_{sol}}, \text{ if } NSAF_{insol} \geq NSAF_{sol} \\ -\dfrac{NSAF_{insol}}{NSAF_{sol}}, \text{if } NSAF_{insol} < NSAF_{sol} \end{cases}$$

where $NSAF_{insol}$ and $NSAF_{sol}$ were the NSAF values at insoluble and soluble fraction, respectively.
