# Supplementary material for: Proteomic Analysis of Bifidobacterium longum subsp. infantis Reveals the Metabolic Insight on Consumption of Prebiotics and Host Glycans
Source: PLoS One. 2013 Feb 26;8(2):e57535. doi: 10.1371/journal.pone.0057535 (PMC3582569; doi:10.1371/journal.pone.0057535)
Supplement: Table S2 — The range of a score that determine the protein location. (PDF) [file pone.0057535.s007.pdf]

Table S2: The range of a score that determine the protein location.

Ave(point)[a]	Location
+0.5 ~ + 1.0	Cell wall association (CWA)
-0.5 ~ -1.0	Cytosol (CYT)
-0.5 ~ +0.5	Unidentified (UI)

[a] The average scores of a protein as determined by the rule in Supplementary Table S1.
