# Supplementary material for: Proteomic Analysis of Bifidobacterium longum subsp. infantis Reveals the Metabolic Insight on Consumption of Prebiotics and Host Glycans
Source: PLoS One. 2013 Feb 26;8(2):e57535. doi: 10.1371/journal.pone.0057535 (PMC3582569; doi:10.1371/journal.pone.0057535)
Supplement: Table S3 — Number of proteins expressed in the B. infantis proteome across different prebiotics. (PDF) [file pone.0057535.s008.pdf]

Table S3: Number of proteins expressed in the *B. infantis* proteome across different prebiotics.

Loction	Carbohydrate						
	Glucose	Lactose	GOS	FOS	Inulin	HMO	Total
Cytosolic (CYT)	140	109	166	104	128	139	196
Cell wall associated (CWA)	85	86	119	143	77	53	93
Unidentified (UI)	165	172	109	100	169	159	251
Total	390	367	394	347	374	351	540
