# Supplementary material for: Proteomic Analysis of Bifidobacterium longum subsp. infantis Reveals the Metabolic Insight on Consumption of Prebiotics and Host Glycans
Source: PLoS One. 2013 Feb 26;8(2):e57535. doi: 10.1371/journal.pone.0057535 (PMC3582569; doi:10.1371/journal.pone.0057535)
Supplement: Table S4 — Linear correlation coefficients (Pearson’s Product) between two proteomes. (PDF) [file pone.0057535.s009.pdf]

Table S4: Linear correlation coefficients (Pearson's Product) between two proteomes.

	LAC	FOS	HMO	MUC	GLC	INL
FOS	0.963					
HMO	0.927	0.913				
MUC	0.888	0.875	0.927			
GLC	0.859	0.842	0.849	0.903		
INL	0.824	0.856	0.792	0.855	0.906	
GOS	0.628	0.631	0.604	0.691	0.881	0.828
