# Supplementary material for: Proteomic Analysis of Bifidobacterium longum subsp. infantis Reveals the Metabolic Insight on Consumption of Prebiotics and Host Glycans
Source: PLoS One. 2013 Feb 26;8(2):e57535. doi: 10.1371/journal.pone.0057535 (PMC3582569; doi:10.1371/journal.pone.0057535)
Supplement: Table S5 — Normalized protein amounts of representative cell surface associated proteins in soluble and insoluble fraction. (PDF) [file pone.0057535.s010.pdf]

Table S5 Normalized protein amounts of representative cell surface associated proteins in soluble and insoluble fraction.

Blon	Name	Soluble fraction (NSAFX10^3)						Insoluble fraction (NSAFX10^3)						Ratio[a] (Fold)					
		Lactose	Glucose	GOS	FOS	Inulin	HMO	Lactose	Glucose	GOS	FOS	Inulin	HMO	Lactose	Glucose	GOS	FOS	Inulin	HMO
1259	Allergen V5/Tpx-1 family protein							9.04±0.10	9.19±0.07	19.05±0.54	14.16±0.38	7.62±0.17	8.18±0.20	Insol	Insol	Insol	Insol	Insol	Insol
2082	lipopolysaccharide biosynthesis	0.53±0.45		0.42±0.00	0.23±0.01	0.21±0.00		2.33±0.18	2.60±0.02	3.95±0.35	3.32±0.05	1.89±0.20	2.05±0.10	4.40	Insol	9.40	14.43	9.00	Insol
2183	Glucose PTS system, IIA subunit		1.98±0.32			2.62±1.52		0.33±0.00	6.78±0.18			16.03±0.42	2.45±0.38	Insol	3.42			6.12	Insol
305	ATP synthase F0, B subunit	0.59±0.00	1.00±0.17	2.78±1.49		0.58±0.00	0.59±0.00	5.39±0.02	4.99±0.04	6.71±0.87		2.07±0.02	2.63±0.84	9.14	4.99	2.41		3.57	4.46
1721	NLPA lipoprotein	3.77±0.29	2.68±0.43	0.67±0.53	3.50±0.68	0.80±0.23	2.12±0.22	6.78±0.03	8.76±0.07	6.06±0.54	4.37±0.07	3.86±0.04	7.39±0.26	1.80	3.27	9.04	1.25	4.83	3.49
2067	Glycosyl transferase, family 51			0.20±0.08				0.30±0.00		3.49±0.07				Insol		17.45			

[a] NSAF ratio between insoluble and soluble fraction (See Supplementary Table S1). ‘Insol’ indicates that the protein was identified only at the insoluble fraction.
