# Supplementary material for: Proteomic Analysis of Bifidobacterium longum subsp. infantis Reveals the Metabolic Insight on Consumption of Prebiotics and Host Glycans
Source: PLoS One. 2013 Feb 26;8(2):e57535. doi: 10.1371/journal.pone.0057535 (PMC3582569; doi:10.1371/journal.pone.0057535)

Supplementary Figure S4 Expression of (a) family 3 and (b) family 5 extracellular solute binding (ESB) proteins. Gray and block bar represent the NSAF values obtained from the proteome of CWA and cytosolic fraction, respectively. LAC; lactose, GLC; glucose, FOS; fructooligosaccharide, INL; inulin, HMO; human milk oligosaccharide, GOS; galactooligosaccharide

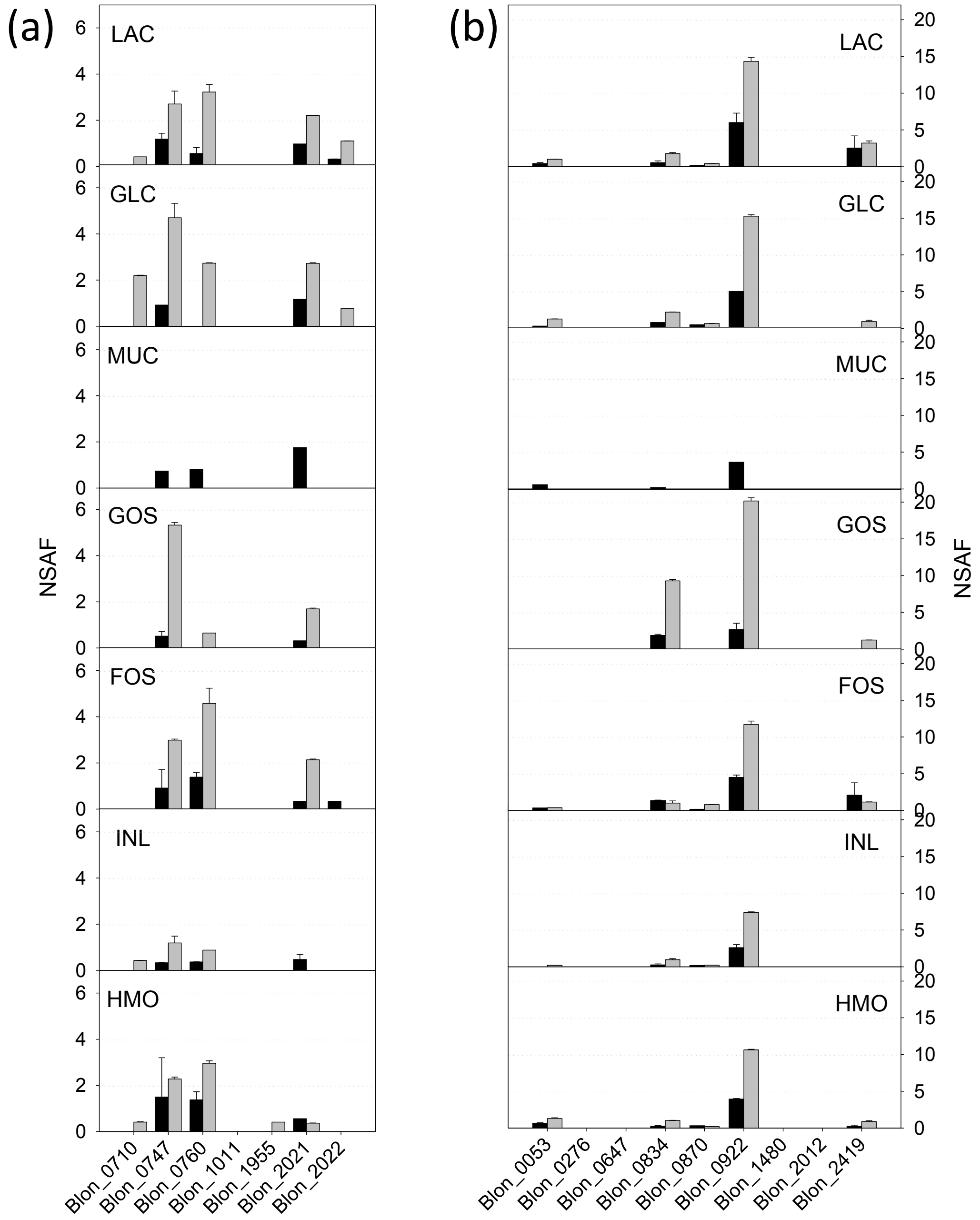

Supplement: Figure S4 — Expression of (a) family 3 and (b) family 5 extracellular solute binding (SBP) proteins. Gray and block bars represent the NSAF values obtained from the proteome of the cell wall associated and the cytosolic fraction, respectively. LAC, lactose; GLC, glucose; FOS, fructooligosaccharides; INL, inulin; HMO, human milk oligosaccharides; MUC, mucin; GOS, galactooligosaccharides. (PDF) [file pone.0057535.s004.pdf]
