# Supplementary material for: Proteomic Analysis of Bifidobacterium longum subsp. infantis Reveals the Metabolic Insight on Consumption of Prebiotics and Host Glycans
Source: PLoS One. 2013 Feb 26;8(2):e57535. doi: 10.1371/journal.pone.0057535 (PMC3582569; doi:10.1371/journal.pone.0057535)
Supplement: Table S6 — List of top 30 most expressed proteins in (a) soluble and (b) insoluble fractions. The rank was determined by the sum of all NSAF values of each carbon sources. The CWA proteins were marked by gray area. Ribosomal proteins were excluded in the list. LAC; lactose, GLC; glucose, GOS; galactooligosaccharides, FOS; fructooligosaccharides, INL; inulin, HMO; human milk oligosaccharides, MUC; mucin. (PDF) [file pone.0057535.s011.pdf]

Table S6 List of top 30 most expressed proteins in (a) soluble and (b) insoluble fractions. The rank was determined by the sum of all NSAF values of each carbon sources. The CWA proteins were marked by gray area. Ribosomal proteins were excluded in the list. LAC; lactose, GLC; glucose, GOS; galactooligosaccharides, FOS; fructooligosaccharides, INL; inulin, HMO; human milk oligosaccharides, MUC; mucin.

Rank	Blon	Name	LAC	GLC	GOS	FOS	INL	HMO	MUC
1	1922	translation elongation factor Tu	33.75	25.97	33.78	46.37	32.68	38.86	30.88
2	694	chaperonin GroEL	24.44	21.21	29.80	31.93	28.01	28.47	32.57
3	679	histone family protein DNA-binding protein	13.55	59.56	30.48	15.00	39.03	13.04	21.44
4	840	L-lactate dehydrogenase	8.22	25.55	31.58	7.37	15.09	21.95	44.43
5	1095	transcriptional regulator, Fis family	16.49	28.02	35.28	13.63	24.97	15.32	15.61
6	1836	Phosphopyruvate hydratase	23.98	20.19	15.32	28.03	14.83	20.35	17.60
7	1696	O-acetylhomoserine/O-acetylserine sulfhydrylase	8.18	11.95	38.73	9.62	21.80	10.12	14.34
8	1722	Fructose-6-phosphate phosphoketolase	17.93	14.46	12.42	17.16	16.71	17.29	15.08
9	1455	homocysteine S-methyltransferase	10.68	14.83	17.26	9.41	25.79	8.55	19.66
10	1096	transketolase	15.78	13.46	18.01	13.62	14.50	12.52	11.29
11	900	glyceraldehyde-3-phosphate dehydrogenase, type I	11.15	15.12	10.64	13.17	20.28	13.41	14.38
12	540	lactaldehyde reductase	3.03	9.11	15.85	5.74	21.86	8.50	8.35
13	2152	phosphoglycerate mutase 1 family	10.28	9.33	15.91	10.26	9.61	8.41	8.51
14	1007	pyridoxamine 5'-phosphate oxidase-related, FMN-binding	8.65	10.98	13.31	8.41	11.40	9.55	10.70
15	2216	adenylate kinase	5.14	12.84	10.64	5.69	10.06	6.10	13.62
16	1745	Pyruvate kinase	8.75	6.50	14.44	8.42	9.54	6.75	6.19
17	1087	Phosphoglycerate kinase	10.04	9.38	6.17	7.30	7.49	7.66	7.41
18	1754	RNA binding S1 domain protein	4.92	8.04	8.48	6.32	7.51	9.27	8.97
19	2191	ribose 5-phosphate isomerase	11.07	7.12	5.63	5.29	7.21	6.04	10.44
20	676	conserved hypothetical protein	10.18	8.95	11.71	6.28	7.90	9.15	5.37
21	309	ATP synthase F1, beta subunit	6.99	6.37	12.91	6.75	6.53	6.21	6.52
22	1731	acetate kinase	6.79	6.50	8.51	7.66	8.18	6.30	7.00
23	1940	succinocarboxamide synthase	9.47	6.98	4.38	7.68	5.71	7.38	9.26
24	1893	glutamine synthetase, type I	12.58	1.92	1.75	15.00	4.53	7.60	3.69
25	1057	translation elongation factor Ts	7.15	5.83	7.54	6.21	7.82	7.60	6.61
26	417	Glucose-6-phosphate isomerase	8.15	5.04	6.47	7.41	5.84	7.74	6.44
27	1923	translation elongation factor G	6.91	7.01	6.24	7.90	6.33	6.32	6.54
28	141	chaperone protein DnaK	4.78	3.92	5.92	8.80	5.07	8.36	6.10
29	1905	Beta-glucosidase	0.72	4.77	13.25	2.13	13.51	1.62	5.47
30	2211	DNA-directed RNA polymerase, alpha subunit	5.51	5.00	5.85	5.54	5.66	5.40	5.33

Blon	Name	LAC	GLC	GOS	FOS	INL	HMO
1922	translation elongation factor Tu	52.38	59.71	52.68	48.01	56.49	57.04
1836	Phosphopyruvate hydratase	32.07	30.10	23.44	28.30	34.51	32.30
694	chaperonin GroEL	33.10	22.32	15.75	33.37	23.15	23.76
840	L-lactate dehydrogenase	20.30	26.65	22.20	10.52	12.63	33.86
1095	transcriptional regulator, Fis family	19.58	17.88	21.53	19.25	21.20	15.98
1096	transketolase	18.81	21.65	10.87	18.68	22.82	19.77
679	histone family protein DNA-binding protein	11.90	5.30	45.10	26.67	10.27	9.86
1722	Fructose-6-phosphate phosphoketolase	16.71	15.08	13.56	11.68	14.53	15.03
2152	phosphoglycerate mutase 1 family	16.49	11.47	18.05	10.94	15.21	11.35
922	ESB protein, family 5	14.33	15.27	20.16	11.71	7.39	10.64
540	lactaldehyde reductase	6.21	10.89	14.16	6.44	22.65	11.46
1259	Allergen V5/Tpx-1 family protein	9.04	9.19	19.05	14.16	7.62	8.18
1745	Pyruvate kinase	8.33	10.34	14.42	7.47	9.89	9.47
2061	ESB protein, family 1				15.51	43.71	0.43
1696	O-acetylhomoserine/O-acetylserine sulfhydrylase	5.69	7.98	12.98	10.76	12.34	5.33
1923	translation elongation factor G	8.60	10.40	9.79	8.40	6.88	10.12
2216	adenylate kinase	9.35	13.19	5.73	7.84	7.66	9.51
1455	homocysteine S-methyltransferase	6.04	6.87	8.79	9.79	9.21	7.57
1715	formate acetyltransferase	4.40	9.93	7.85	4.47	10.87	10.29
676	conserved hypothetical protein	9.68	9.39	13.60	4.68	9.92	
900	glyceraldehyde-3-phosphate dehydrogenase, type I	6.91	10.44	6.80	6.04	9.95	5.66
1905	Beta-glucosidase	1.51	3.48	11.75	4.24	20.21	2.27
1893	glutamine synthetase, type I	7.76	2.95	3.16	15.71	7.82	5.95
1940	succinocarboxamide synthase	6.49	8.83	6.03	7.18	5.47	8.68
1731	acetate kinase	7.51	8.09	6.95	4.93	9.58	5.43
2475	ABC transporter related	1.86	3.11	7.34	11.66	10.77	7.59
417	Glucose-6-phosphate isomerase	6.77	5.54	6.76	7.94	7.99	5.14
1164	Phosphoribosylaminoimidazolecarboxamide formyltransferase	6.38	9.24	4.46	5.63	7.00	6.49
309	ATP synthase F1, beta subunit	4.73	6.51	9.97	5.04	6.42	4.88
1721	NLPA lipoprotein	6.78	8.76	6.06	4.37	3.86	7.39
